# Molecular Characterization of Chitin Synthase Gene in *Tetranychus cinnabarinus* (Boisduval) and Its Response to Sublethal Concentrations of an Insecticide

**DOI:** 10.3390/insects12060501

**Published:** 2021-05-28

**Authors:** Tianrong Xin, Zhenzhen Li, Jia Chen, Jing Wang, Zhiwen Zou, Bin Xia

**Affiliations:** School of Life Sciences, Nanchang University, Nanchang 330031, China; xintianrong@ncu.edu.cn (T.X.); l1245584341@163.com (Z.L.); cchen223@ford.com (J.C.); wangjingyn@126.com (J.W.); zouzhiwen@ncu.edu.cn (Z.Z.)

**Keywords:** *Tetranychus cinnabarinus*, chitin synthase, transcript expression, insecticide exposure

## Abstract

**Simple Summary:**

In this study, we identified chitin synthase 1 gene (*TcCHS1*) from *Tetranychus cinnabarinus* (Boisduval) and then explored the gene expression levels of *TcCHS1* at different developmental stages of *T. cinnabarinus*. We also investigated the effects of sublethal concentrations of diflubenzuron on the toxicities and survivals of *T. cinnabarinus* eggs and larvae as well as *TcCHS1* expression levels. Our results demonstrated that *TcCHS1* was essential for growth and development, and diflubenzuron exposure affected chitin metabolism. This work was undertaken to establish a foundation for further research on the functions of chitin synthase. It will provide a new target for controlling of *T. cinnabarinus* in the agricultural ecosystem.

**Abstract:**

The carmine spider mite, *Tetranychus cinnabarinus* (Boisduval), is one of the most important acarine pest species. At present, its control remains primarily dependent on using various chemical insecticides/acaricides in agricultural crops worldwide. To clarify the mechanism whereby *T. cinnabarinus* responds to insecticide exposure, we identified the chitin synthase 1 gene (*TcCHS1*) and then explored the gene expression levels of *TcCHS1* at different developmental stages of *T. cinnabarinus*. We also investigated the effects of sublethal concentrations of diflubenzuron on the toxicities and survivals of *T. cinnabarinus* eggs and larvae as well as *TcCHS1* expression levels. The full-length cDNA sequence contains an open reading frame (ORF) of 4881 nucleotides that encoded for a 1474 amino acid residues protein. The predicted TcCHS1 protein had a molecular mass of 168.35 kDa and an isoelectric point of 6.26, and its amino acid sequence contained all the signature motifs (EDR, QRRRW and TWGTR) of chitin synthases. The results of phylogenetic analyses demonstrated that the putative CHS1 amino acid sequence of *T. cinnabarinus* revealed high similarities with chitin synthases in other insects and mites. Additionally, at the molecular level, transcriptional analysis by real-time quantitative PCR in different developmental stages of *T. cinnabarinus* revealed that *TcCHS1* mRNA was expressed in all stages, and highest in eggs and female adults, but lowest in deutonymphs. Furthermore, the results of toxicity bioassays indicated that diflubenzuron treatment resulted in high mortality rates in eggs and larvae of *T. cinnabarinus*. The mRNA expression levels of *TcCHS1* from the eggs and larvae of *T. cinnabarinus* were up-regulated in response to sublethal concentrations of diflubenzuron exposures. Together, all these results demonstrate that diflubenzuron has ovicidal and larvicidal effects and *TcCHS1* may play an important role in the growth and development of *T. cinnabarinus* and may disrupt the chitin biosynthesis, thereby controlling *T. cinnabarinus* populations.

## 1. Introduction

Insects must periodically undergo several molting processes in order to allow growth and development, and during the molting process, they digest the old cuticle and produce the new one by chitin degradation and synthesis simultaneously [1]. Proverbially, chitin is a major essential component of the exoskeletons and peritrophic membranes of insects and mites and crucial for their growth and development by manipulating chitin degradation and synthesis [2,3]. Chitin synthase (CHS) is the vital and last enzyme of the chitin biogenesis pathway in insects, belongs to the family of 2 β-glycosyltransferases, and is an integral membrane protein that transports chitin outside the membranes of insect cells [1]. In 2000, the first insect chitin synthase from the sheep blowfly *Lucilia cuprina* was isolated and sequenced [4]. Since then, the chitin synthases of many insects and mites have been cloned and characterized, such as *Aedes aegypti* [5], *Drosophila melanogaster* [6], *Anopheles quadrimaculatus* [7], *Spodoptera exigua* [8], *Locusta migratoria manilensis* [9], *Ostrina funracalis* [10], *Anopheles gambiae* [11], *Leptinotarsa decemlineata* [12], *Panonychus citri* [13], and *Tetranychus urticae* [14]. These results revealed that the *CHSs* are usually classified into CHS1 (CHS-A) and CHS2 (CHS-B) [15]. CHS1 is primarily responsible for chitin formation in the exoskeletal cuticle and tracheae, while CHS2 participates in chitin synthesis in insect midgut peritrophic membrane (PM) that blankets food bolus to facilitate digestion [16,17,18]. CHS1 expression of *Tribolium castaneum* was different in the developmental stages and highest in the prepupal to young pupal stages; this result is consistent with a major role for this gene in chitin synthesis associated with pupal and adult cuticles [16]. Bansal et al. reported that chitin synthase from the soybean aphid *Aphid glycines* was expressed in different developmental stages and all major tissues including gut, fat body and integument [17]. The results of development-specific expression patterns by Xia et al. indicated that the CHS1 of *Panonychus citri* was constantly expressed in all stages but highest expressed in the eggs [13]. Furthermore, the recent reports about gene functional analyses of the CHS gene using RNA interference (RNAi) in different insect species have indicated that the knockdown of *LdCHS* in *Leptinotarsa decemlineata* severely affected larval growth, caused larval lethality and impaired larval-larval molting, larval-pupal ecdysis and adult emergence [12]. The silencing of the CHS1 gene in white-backed planthopper *Sogatella furcifera* nymphs resulted in malformed phenotypes and killed most of the treated nymphs [19]. Together, chitin biosynthesis is essential for arthropod growth and development, and the CHS of the chitin biosynthesis pathway represents a potentially desirable target for developing a new strategy for arthropod pest management in agricultural systems because chitin is absent in plants and vertebrate animals [16]. 

Benzoyphenyl ureas (BPUs) belong to chitin synthesis inhibitors (CSIs) used as insecticides and are grouped in Insecticide Resistance Action Committee (IRAC) class 15, seriously interfering with the chitin biosynthesis of pests, leading to failure of molting and development due to their unique mode of actions different from traditional broad-spectrum insecticides [20]. Merzendorfer reported that triflumuron was a benzoylphenylurea which could lead to molting failure, deformity and even death of pests by interfering with the entomic chitin biosynthesis pathway [1]. Mahdy et al. found that the treatment with different concentrations of lufenuron (CSIs) against the fifth nymphal instar females of the desert locust *Schistocerca gregaria* resulted in the failure of ecdysis to adults [21]. Diflubenzuron (DFB), a kind of benzoylphenylurea-selective insecticide with low toxicity, interferes with chitin formation in the procuticle and the deposition of epicuticle, resulting in abortive molting and other alterations in the physiological processes of various arthropod species. It is studied extensively in agricultural and forestall systems against numerous pests [22,23,24,25,26]. In a previous study performed by Zhang and Zhu, the result revealed that *Anopheles quadrimaculatus* larvae that were exposed to diflubenzuron had significantly increased CHS1 expression levels connected with decreased chitin synthesis, which may imply a possible inhibition of chitin synthase or abnormal CHS translocation [7]. Lu et al. reported that the relative expression level of chitin synthase was significantly upregulated after diflubenzuron exposure to fifth-instar nymphs of Asian citrus psyllid *Diaphorina citri* at 24 h and 48 h [27]. Moreover, a recent study revealed that the resistance mechanism to diflubenzuron included a target-site mutation in the gene chitin synthase 1 between insects and mites [26]. In general, the application of diflubenzuron can affect chitin biosynthesis by chitin synthase; however, because chitin biosynthesis is a complex process involving different catalytic enzymes and is associated with complex pathways, at present, the molecular mode of action of diflubenzuron has been discussed for several decades and is still not fully understood. Merzendorfer et al. utilized genomic and proteomic approaches to study the effects of diflubenzuron in a model beetle species and stored product pest *Tribolium castaneum* (red flour beetle); their results suggested that the expression levels of genes encoding chitin synthases, chitin deacetylases and chitinolytic enzymes were not significantly altered in response to diflubenzuron [28]. Similarly, another study demonstrated that chlorfluazuron did not change the expression of CHS genes in *Plutella xylostella* larvae [29]. Abo-Elghar et al. revealed that the target molecule of diflubenzuron in the inhibition of chitin synthesis in insects may be the sulfonylurea receptor (SUR) because diflubenzuron is structurally similar to sulfonylureas [30]. Moreover, in *Tribolium castaneum* [28] and *D. melanogaster* [23], diflubenzuron does not affect mRNA expression of CHS and does not act directly on the catalytic step of chitin synthesis [31]. It even blocks the chitin synthesis of the *stable fly* [22]. 

The carmine spider mite, *Tetranychus cinnabarinus* (Boisduval) (Acari: Tetranychidae), is considered as the red form or a synonym of *Tetranychus urticae* [32]; it is a major polyphagous pest species with a worldwide distribution and a wide range of host plants [33]. Thus, its effective control and management are of great significance. Many efforts were made to control *T. cinnabarinus* in agriculture. However, currently, the control of this pest remains to largely rely on using a range of synthetic insecticides/acaricides in field crops in many countries worldwide. Consequently, *T. cinnabarinus* has developed serious resistance to the majority of various insecticides/ acaricides [34], so its effective control is a continuing challenge. 

Understanding the responses of diflubenzuron exposure is an important part of the metabolic mechanism of *T. cinnabarinus*. Currently, little is known regarding the effects of diflubenzuron on the biological traits as well as chitin synthase of *T. cinnabarinus*. Therefore, in the present study, the toxicological analysis and survival of *T. cinnabarinus* eggs and larvae responding to diflubenzuron exposure were determined. Moreover, the chitin synthase gene was cloned, its bioinformatics characteristics were analyzed and the mRNA expression profiles of chitin synthase were assessed at different developmental stages of *T. cinnabarinus*. Finally, the expression levels of the chitin synthase gene were estimated in order to explore the potential mechanism of *T. cinnabarinus* exposed to sublethal concentrations of diflubenzuron. All the results obtained in this study might provide a further understanding of the mechanism contributing to the adaptation of *T. cinnabarinus* to BPUs and will lay the foundation for the effective management of *T. cinnabarinus*. 

## 2. Material and Methods

### 2.1. Mass Rearing of Experimental Mites

The laboratory population of *T. cinnabarinus* was initiated with individuals collected from pesticide-free cassava plant leaves in a Cassava Plantation, Dongxiang County, Jiangxi Province, China, and then were continuously cultured at the School of Life Sciences, Nanchang University, since 2010. The colony was continually grown on *Phaseolus vulgaris* seedlings without exposure to any insecticides in a greenhouse under approximately 25 ± 2 °C and 60 ± 10% relative humidity and 14:10 h photoperiod (Light: Dark). 

### 2.2. Preparation of Eggs and Larvae of T. cinnabarinus Synchronized in Development

With the aim to produce large cohorts of eggs and larvae of *T. cinnabarinus* that coordinated in their development, respectively, the procedures were detailed as follows: approximately 20 adult female mites (3 to 5 days old) of *T. cinnabarinus* were transferred onto each fresh detached *P. vulgaris* leaf disc (about 4 cm^2^) which was placed on water-saturated filter-paper and sponge in the petri dish (8 cm diameter, 1 cm high), and allowed to lay eggs for 24 hours in an artificial climate chamber at 25 ± 1 °C and 60 ± 10% relative humidity and 14:10 h photoperiod (Light: Dark). After 24 h, these adult females were removed with a soft brush without damaging the eggs. A portion of these 1-day-old eggs was subjected to the following bioassay or sublethal effects of experiments; another portion of eggs was incubated until larvae emergence. These larvae (1-day-old) were considered as developmentally synchronized mites which were used in the following bioassay or sublethal effects of experiments. 

### 2.3. Diflubenzuron Bioassays and Sublethal Effect of Diflubenzuron against Developmentally Synchronized Eggs and Larvae of T. cinnabarinus

In this experiment, the insecticide was diflubenzuron (purity > 98%) which was purchased from Sigma-Aldrich (St. Louis, MO, USA). 1 mg diflubenzuron was dissolved with 1 mL of acetone, to prepare 1 mg/mL of the stock solution. Then the stock solution was diluted to seven different concentrations in distilled water for the bioassay or treatment of the synchronous cohorts of eggs and larvae of *T. cinnabarinus*, respectively. 

In our study, the diflubenzuron bioassays against eggs and larvae of *T. cinnabarinus* were determined using the previously described leaf-disc dipping methodology with slight modification [35]. The developmentally synchronized eggs and larvae were collected as described above for the experimentation; subsequently, each *P. vulgaris* leaf disc with these eggs or larvae were individually dipped for 10 s into seven serially diluted concentrations of diflubenzuron covering the range of 0–100% mortality, were dried with a filter in the toxicity tests, then all mites in the petri dish were incubated in an artificial climate chamber at 25 ± 1 °C and 60 ± 10% relative humidity and 14:10 h photoperiod (Light: Dark). The experiment was repeated four times using three replicates for each concentration. After 24 h, the survival of individual larvae was determined by touching the body of each mite with a soft brush under a stereo-microscope (S9i, Leica, Germany); those unable to move were considered as dead. The eggs were considered dead if they could not successfully hatch into larvae. According to the experimental data obtained from the bioassay, final mortality was calculated following Abbott’s formula [36]: percent corrected mortality = [(mortality in treatment group − mortality in control group)/(1 − mortality in the control group)] × 100%. The mortalities of the control group never exceeded 10%. The results were expressed as the means ± standard error of the means. The virulence regression equation, half lethal concentration LC_50_, 95% confidence interval, card square value and correlation coefficient were performed by probit regression analysis using the SPSS 20.0 statistical software for Windows. At the same time, the corresponding sublethal concentration (LC_10_, LC_20_, LC_30_, LC_40_ and LC_50_) of diflubenzuron against eggs and larvae of *T. cinnabarinus* were also obtained (data are shown in Table 3).

In order to assess the sublethal effects of diflubenzuron on eggs and larvae of *T. cinnabarinus* under laboratory conditions, we still used leaf-dipping methodology as described above to treat developmentally synchronized eggs and larvae. Each detached *P. vulgaris* leaf with eggs or larvae was dipped in their corresponding sublethal concentrations (LC_10_, LC_20_, LC_30_, LC_40_ and LC_50_) of diflubenzuron for 10 s, respectively. Eggs or larvae treated with distilled water were used as the control. After 24 h, a portion of these alive individuals was collected and immediately frozen in liquid nitrogen for total RNA extraction in the following molecular experiments; another portion of mites was continuously reared, checked and recorded daily until larvae or protonymphs emergence. Approximately 200 eggs or 200 larvae were contained in each treatment. At least three biological replicates were conducted for each treatment. 

### 2.4. Cloning and Sequencing of Chitin Synthase cDNA of T. cinnabarinus

Total RNA was extracted from the whole body of *T. cinnabarinus* larvae using the Eastep^®^ Super Total RNA Extraction Kit (Shanghai Promega Trading Co., Ltd., Shanghai, China) in accordance with the manufacturer’s instructions. The concentration and purity of total RNA in this study were estimated by ultramicro-spectrophotometers NanoDrop2000 (Thermo Fisher Scientific, Lithuania, USA) at the absorbance ratios of A260/230 and A260/280. The integrity of the RNA sample was examined via 1% agarose gel electrophoresis. The total RNA sample was stored at -80°C ultra-low temperature freezer after dissolution in RNase-free water. Subsequently, the first strand of cDNA was synthesized from 2 μg of total RNA using the FastQuant RT Kit (with gDNase) (Tiangen, Beijing, China). All the first-strand cDNA templates were maintained in a −20 °C refrigerator until they were used in the subsequent experiment. The 5’ and 3’ RACE cDNAs were performed via the SMARTer^®^ RACE cDNA Amplification Kit (Clontech, CA, USA).

Two pairs of the nest and degenerate primers were designed according to the reported conserved sequences of CHS cDNA of the other insects and mites; they were used to amplify the partial fragments of the *TcCHS1* gene by polymerase chain reaction (PCR). The 5′ and 3′ end sequences of the *TcCHS1* gene were obtained by the rapid amplification of cDNA ends (RACE) PCR. The PCR reaction system contained 25 μL reaction mixtures including 1 μL cDNA sample, 1 μL of each pair of primer, 4 μL dNTP, 2.5 μL 10 × buffers and 0.25 μL DNA polymerase. The PCR procedure was initially 94 °C for 3 min, followed by 35 cycles of 94 °C 30 s, 50–55 °C 30 s and 72 °C for 30 s, then maintained at 72 °C for 10 min. In the study, all the products of PCR and RACE were individually purified and ligated into the pGEM-T Easy vector (Shanghai Promega Trading Co., Ltd., Shanghai, China); subsequently, its ligation mixture was transformed into *E. coli* DH5α complete cells. At last, these products were sent to Sangon Biotech (Shanghai, China) for sequencing. All the specific and degenerate primers which were used in the experiment are presented in Table 1.

### 2.5. Bioinformatics and Phylogenetic Analysis of TcCHS1

The full-length cDNA sequence of the *TcCHS1* gene was obtained by assembling all the sequencing results with SeqMan software 6.0. Similarities of all the sequences were searched using the non-redundant protein sequence (nr) database of the National Centre for Biotechnology Information (NCBI) website. The cDNA sequence was translated into the corresponding deduced amino acid sequences. The complete open-reading frames (ORF) of *TcCHS1* were subsequently confirmed with the ORF Finder program at the NCBI website. The physical and chemical properties of the *TcCHS1* amino acid sequence including molecular formula and weight, isoelectric point and so on and were deduced via a proteins online analysis tools. The signal peptide of the protein was obtained via the SignalP 3.0 online server program, and the conserved domain of the protein was performed using the SMART Online program. The TMHMM v.2.0 program was used to analyze all the transmembrane helices.

To reveal the evolutionary relationship of CHS orthologs, a phylogenetic tree was constructed based on the deduced amino acid sequences from other insects and mites with 1000 bootstrap replicates in MEGA 5.05 software. The optimal amino acid substitution model WAG+G was used basing AIC calculations on jModelTest 2.1.4 and the corresponding phylogenetic tree was constructed on the basis of the maximum likelihood (ML) method. 

### 2.6. mRNA Expression Analysis of the Chitin Synthase Gene of T. cinnabarinus

The mRNA expression levels of the chitin synthase gene of *T. cinnabarinus* at different developmental stages and different sublethal concentrations of diflubenzuron exposure to eggs and larvae of *T. cinnabarinus* were individually determined by reverse transcription, quantitative, real-time PCR (RT-qPCR), which was used on a StepOnePlus Real-Time PCR System (Thermo Fisher Scientific, Lithuania, USA) using SYBR Green Mix. In brief, total RNA was extracted from the whole body of each sample using the Eastep^®^ Super Total RNA Extraction Kit (Shanghai Promega Trading Co., Ltd., Shanghai, China) in accordance with the manufacturer’s protocols. The quality and quantity of total RNA for each sample were evaluated on 1% gel electrophoresis and a NanoDrop 2000 spectrophotometer (Thermo Fisher Scientific, Lithuania, USA). Genomic DNA was removed using RQ-Free DNAase (Shanghai Promega Trading Co., Ltd., Shanghai, China). A unit of 3 μg of each total RNA was used for the synthesis of cDNA by using FastQuant RT Kit (Tiangen, Beijing, China). The gene-specific primer pairs TC-CHS-F and TC-CHS-R were designed according to the full length of *TcCHS1* in Primer Premier 5.0 software (Table 1). The primers of β-actin-F and β-actin-R from *T. cinnabarinus* were designed according to the cDNA sequences of β-actin (Table 1). β-actin was used as the reference gene. The RT-qPCR was performed with 20 μL reaction mixtures which contained 2 μL cDNA templates, 0.4 μL primers (10 μmol/L primer) and SYBR Green I. Each RT-qPCR reaction in this study was as follows: at 94 °C for 3 min for pre-denaturation, followed by 36 cycles of denaturation at 94 °C for 30 s, 57 °C for 30 s and elongation at 72 °C for 1 min, then maintained at 72 °C for 10 min. At last, the melt curve analyses in RT-qPCR analysis were inserted into each reaction at 65–95 °C for every 10 s and slowly increased by 0.5 °C. All the groups in the experiment were conducted with three independent biological replicates and three technical replicates. The relative expression level of the chitin synthase gene was calculated using the 2^−ΔΔCt^ method [37]. 

### 2.7. Statistical Analysis

All the statistical analyses of the data were performed with the SPSS 20.0 software (IBM Corporation, USA). The results were calculated from the mean values of the three independent biological replicates. The mortality ad mRNA expression levels of chitin synthase were analyzed by one-way analysis of variance (ANOVA) followed by Duncan’s test to determine the significance of differences. The levels of significance were separated by *p*-value < 0.05.

## 3. Results

### 3.1. The Toxicities of Diflubenzuron against Eggs and Larvae of T. cinnabarinus

The median lethal concentration (LC_50_) values of the eggs and larvae were 15.825 and 16.373 mg/L, respectively (Table 2). These results revealed that diflubenzuron had higher toxicity against eggs compared to larvae of *T. cinnabarinus*. 

### 3.2. Effects of Sublethal Concentrations of Diflubenzuron on the Survival Rate in T. cinnabarinus Eggs and Larvae

The average survival percentage in eggs gradually decreased when *T. cinnabarinus* eggs were treated with higher concentrations of diflubenzuron (LC_10_-LC_50_) (Table 3), comparing with the control. No statistically significant effect of diflubenzuron was observed on the eggs’ survival in the LC_10_-treated group, compared with the control (*p* < 0.05), while there were statistically significant differences among LC_10_-, LC_30_- and LC_40_ (or LC_50_)-treated groups (*p* < 0.05) (Table 4). 

**Figure 1:**
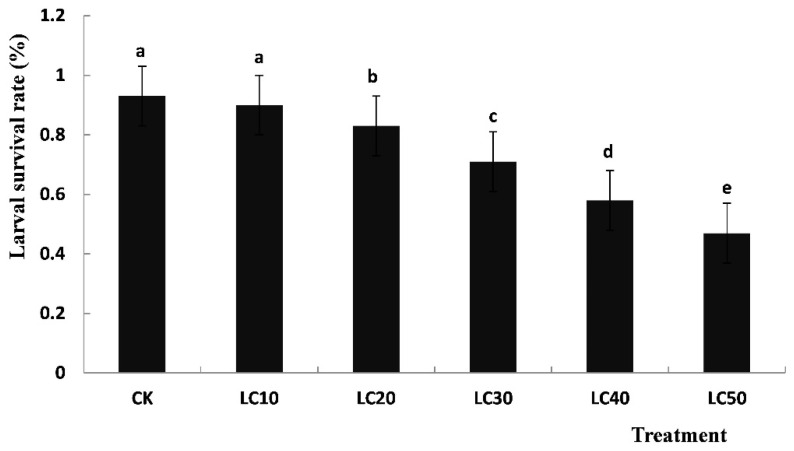
Survival rates of T. cinnabarinus larvae treated with a sublethal concentration of diflubenzuron. Notes: CK served as the control group. The data are expressed as means ± SE of three replicates and subjected to one-way ANOVA followed by Duncan’s multiple range test. Different letters above each bar indicate a statistically significant difference at *p* < 0.05; same lowercase letters indicate non-significant changes.

As shown in Figure 1, the larval survival rates of all the sublethal concentrations of diflubenzuron-treated groups in *T. cinnabarinus* were lower than that of the control and they gradually decreased with the increasing concentrations of diflubenzuron. The larval survival rate in the LC_50_-treated group was the lowest: only 50.54% of the control group. A statistically significant difference (*p* < 0.05) was found among the different sublethal concentrations (LC_10_, LC_20_, LC_30_, LC_40_ and LC_50_) of diflubenzuron-treated groups, whereas no significant difference was observed between the control and LC_10_-treated groups (*p* < 0.05): the survival rate of the control group was about 93%, while it was 90% in the LC_10_-treated group. 

### 3.3. Identification and Characterization of Chitin Synthase Gene from T. cinnabarinus

**Figure 2:**
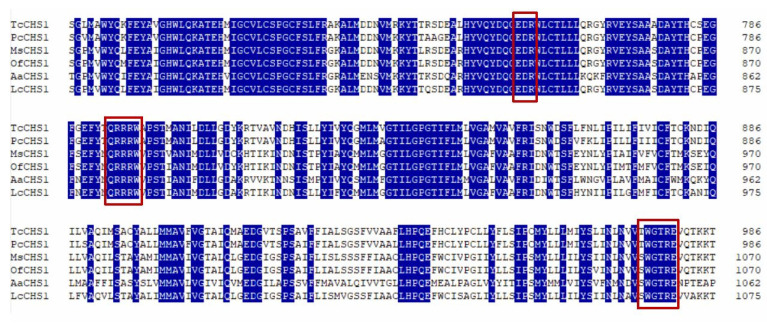
Alignment of putative-conserved catalytic domains of chitin synthase genes from two mite species and four insect species are suggested. The following insect and mite chitin synthase sequences were used in the alignment: *Tetranychus cinnabarinus* (Tc, KM242062), *Panonychus citri* (Pc, KM242063), *Manduca sexta* (Ms, AY062175), *Ostrinia furnacalis* (Of, EU376026), *Aedes aegypti* (Aa, AF223577), and *Lucilia cuprina* (Lc, AF221067). Numbers on the right side represent amino acid positions. The chitin synthase signature sequences “EDR”, “QRRRW” and “TWGTR” in the domains of the deduced amino acid sequences are indicated in red boxes, respectively.

In the current study, the full-length *TcCHS1* sequence was 4881 bp (GenBank accession in NCBI is KM242062) and had an ORF of 4425 bp, which encoded a protein of 1474 amino acid residues with a predicted mass of approximately 168.35 kDa and an isoelectric point of 6.26, with a 5′-untranslated region (UTR) of 198 bp and a 3′-UTR of 258 bp. Based on the deduced amino acid sequence encoded by the *TcCHS1* gene, it was predicted there would be a typical transmembrane protein with seventeen transmembrane helixes. There were three domains (A, B and C) in the deduced amino acid sequence encoded by the *TcCHS1* gene. Domain A was located in the N-terminal and contained 606 amino acids with 10 transmembrane helixes. There were 299 amino acids with five transmembrane helixes containing the chitin synthase signature sequences “EDR”, “QRRRW” and “TWGTR” in the key domain B of the deduced amino acid sequence (Figure 2). Domain C was located in the C-terminal and contained 568 amino acids. O-glycosylation sites of the deduced amino acid sequence of chitin synthase in *T. cinnabarinus* were NRTG, NLSD, NLST, NITF and NSSG. Signal peptide prediction of the deduced amino acid sequence of chitin synthase in *T. cinnabarinus* suggested that there was no signal peptide site, indicating that this protein encoding the *TcCHS1* gene was not excretive. Alignment of the deduced amino acid sequence of *TcCHS1* and those of other insects demonstrated that it shared 98% and 55% similarity with that of *T. urticae* and *Metaseiulus occidentalis,* respectively, and it revealed approximately 50% similarity with those of other insects. 

**Figure 3:**
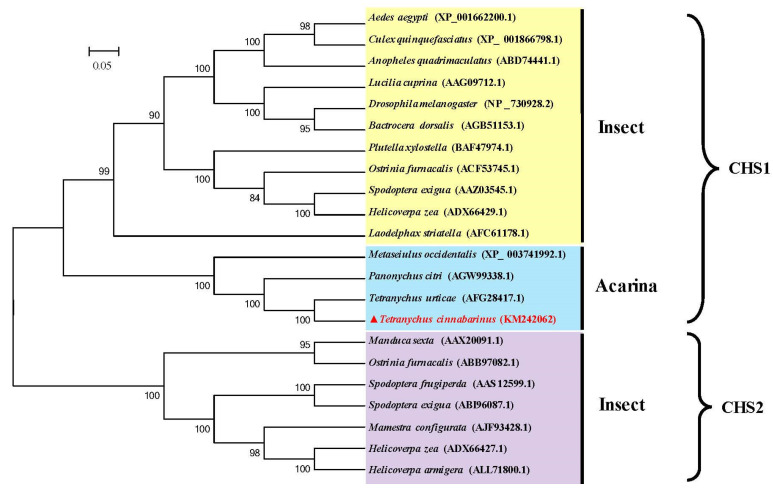
Phylogenetic relationships of chitin synthase of *T. cinnabarinus* with those of other arthropod species based on the deduced amino acid sequences alignment. The phylogenetic tree was generated by MEGA 5.05 using the maximum likelihood (ML) method. Numbers above the branches are bootstrap support values. The completely assembled and annotated *TcCHS1* sequence was deposited in the National Center for Biotechnology Information (NCBI) GenBank under accession number KM242062. Sequences were taken from GenBank database (GenBank accession number in parentheses). *TcCHS1*, *Tetranychus cinnabarinus* (KM242062); *TuCHS1*, *Tetranyus urticae* (AFG28417.1); *PcCHS1*, *Panonychus citri* (AGW99338.1); *MeCHS1*, *Metaseiulus occidentalis* (XP-003741992.1); *AaCHS1*, *Aedes aegypti* (XP-00166200.1); *CqCHS1*, *Culex qinguefasciatus* (XP-001866798.1); *AqCHS1*, *Anopheles quadrimscalatus* (ABD74441.1); *LcCHS1*, *Lucilia cuprina* (AAG09712.1); *DmCHS1*, *Drosophila melanogaster* (NP-730928.2); *BdCHS1*, *Bactrocera dosalis* (AGB51153.1); *PxCHS1*, *Plutella xylostella* (BAF47974.1); *OfCHS1*, *Ostinia furnacalis* (ACF53745.1); *SeCHS1*, *Spodoptera exigua* (AAZ03545.1); *HzCHS1*, *Helicoverpa zea* (ADX66429.1); *LsCHS1*, *Laodelphax striatella* (AFC61178.1); *MsCHS2*, *Manduca sexta* (AAX20091.1); *OfCHS2*, *Ostrinia fumacalis* (ABB97082.1); *SfCHS2*, *Spodoptera frugiperda* (AAS12599.1); *SeCHS2*, *Spodoptera exigua* (ABI96087.1); *McCHS2*, *Mamestru configurata* (AJF93428.1); *HzCHS2*, *Helicoverpa zea* (ADX66427.1); *HaCHS2*, *Helicoverpa armigera* (A LL71800.1).

The phylogenetic analysis revealed that the CHS proteins of all the insect and mite species were clearly separated into two clades including CHS1 and CHS2; moreover, the CHS1 clade was separated into two subclades containing insects and mites, with *TcCHS1* belonging to the mite CHS1 protein subclade. Additionally, the putative CHS amino acid sequence of *T. cinnabarinus* showed a high similarity with the sequences of CHS1 in other insects and was most closely related to that of *T. urticae* (Figure 3).

### 3.4. The Different Developmental Expression Patterns of TcCHS1

**Figure 4:**
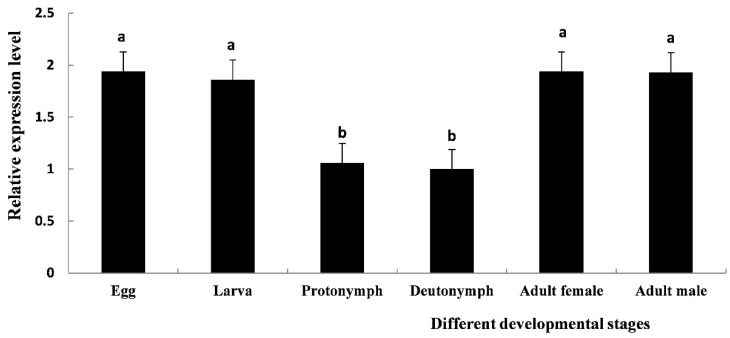
The relative mRNA expression level of *TcCHS1* at different developmental stages of *T. cinnabarinus* was determined by real-time quantitative PCR (RT-qPCR), including eggs, larvae, protonymphs, deutonymphs, adult females and adult males. The *β-actin* gene of *T. cinnabarinus* was used as a reference gene. Each value is represented as the mean (± SE) of four replications. Means within the same column followed by the same lowercase letter(s) are not statistically significant different using Duncan’s test at a 95% level of confidence (*p* < 0.05).

*TcCHS1* was expressed at six different developmental stages including eggs, larvae, protonymphs, deutonymphs, adult males and adult females (Figure 4). The relative expression levels of *TcCHS1* at different developmental stages showed the following trends: eggs > adult females > larvae > adult males > protonymphs > deutonymphs. The expression levels of the eggs and adult females were the highest, which were 1.935 times higher than that of the deutonymphs. The expression level of the larvae was 1.929 times that of the deutonymphs, and the expression levels of the adult males and the protonymphs were 1.863 and 1.062 times that of the deutonymphs.

### 3.5. Effect of Sublethal Concentrations of Diflubenzuron Exposure on TcCHS1 Gene Expression in Eggs and Larvae of T. cinnabarinus

**Figure 5:**
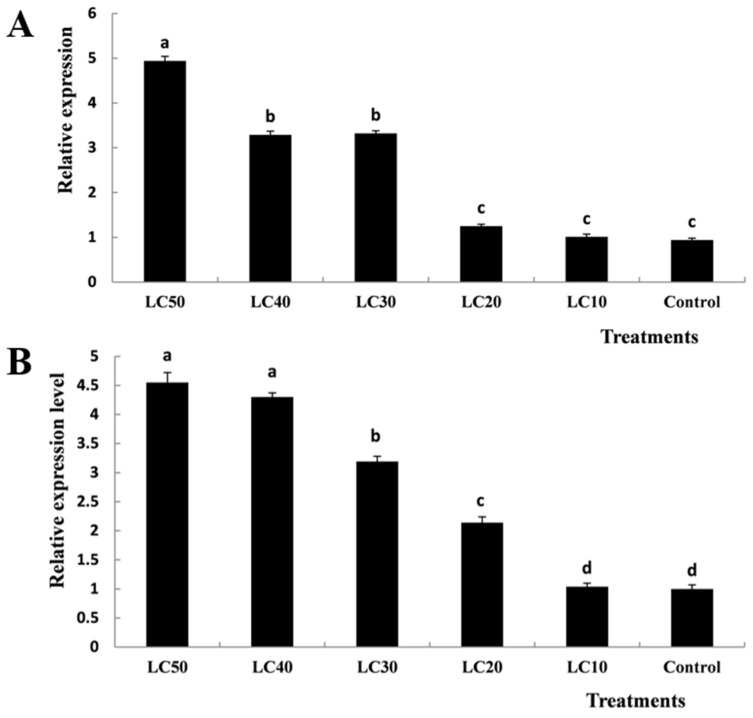
Effects of different concentrations of diflubenzuron on the mRNA relative expression levels in *T. cinnabarinus* eggs (**A**) and larvae (**B**). Bar graphs represent means ± SE; different lowercase letters on each bar indicate significant differences (*p* < 0.05).

The current results revealed that the relative expression levels of the *TcCHS1* gene in *T. cinnabarinus* eggs gradually increased with the increasing of concentration of diflubenzuron and peaked in the LC_50_-treated group, compared with the control (Figure 5A). Although no statistically significant differences were observed between the LC_40_-treated and LC_30_-treated groups as well as between the low concentration treatment (LC_20_ and LC_10_) groups and the control (*p* < 0.05), the statistically significant differences of mRNA expression levels of *TcCHS1* were observed among high-concentration (LC_50_), medium-concentration (LC_40_ and LC_30_) and low-concentration (LC_20_ and LC_10_) treatments (*p* < 0.05). 

Similarly, the relative expression levels of the *TcCHS1* gene in *T. cinnabarinus* larvae gradually increased with the increasing of concentration of diflubenzuron and peaked in the LC_50_-treated group, compared with the control (Figure 5B). Statistically significant differences were observed in the LC_50_, LC_30_, LC_20_ and LC_10_ treatment groups (*p* < 0.05). However, there were no statistically significant differences between the LC_50_-treated and LC_40_-treated groups as well as the LC_10_-treated group and the control (*p* < 0.05). 

## 4. Discussion

The molting during metamorphosis of arthropods is a complex process containing a new cuticle forming and an old one degrading, simultaneously [38]. Several studies have demonstrated that chitin synthases (CHSs) catalyze the polymerization of N-acetylglucosamine into chitin in the final step of the chitin biosynthetic pathway, so they play crucial roles in the process of the arthropod’s growth and development [27]. CHSs are split into CHS1 (CHS-A) and CHS2 (CHS-B) based on their differences of deduced amino acid sequence similarity, distribution and biological function [4]. CHS1 is responsible for the chitin synthesis of the cuticle, whereas CHS2 is specialized for the synthesis of chitin in the peritrophic membrane (PM) of the insect midgut [39]. In this study, the full-length cDNA of the chitin synthase gene *T. cinnabarinus* was cloned and characterized; the result of amino acid sequence alignment and phylogenetic analysis of insect and mite species revealed that all the chitin synthase genes were divided into two clear clades including CHS1 and CHS2, and the chitin synthase of *T. cinnabarinus* was closely related with that of the mite CHS1. Moreover, the result of this study demonstrated that *TcCHS1* cDNA was 4881 bp in length with an open reading frame of 4425 bp that encoded a putative protein of 1474 amino acids with a predicted pI of 6.26; this slightly acidic pI was conducive to its biological function in the cuticle [19]. In addition, our results indicated that *TcCHS1* was predicted to have a molecular mass of 168.35 kDa protein which contained 17 transmembrane helixes, so it is similar to the CHS1 protein of other insect and mite species [27]. Furthermore, consistent with the CHS1 proteins of other arthropods, *TcCHS1* contained the signature motifs “EDR”, “QRRRW” and “TWGTR” which were found in all chitin synthases and considered to be crucial for the catalytic mechanism [39]. Among 17 transmembrane helixes, five were located in the key catalytic domain and formed a topological feature named the five transmembrane span (5-TMS) regions which were present in all chitin synthases of arthropods [7]. 

In the current study, the developmental stage-dependent expression patterns of the chitin synthase gene of *T. cinnabarinus* were described by real-time quantitative PCR. The results revealed that *TcCHS1* was expressed at different developmental stages of *T. cinnabarinus* including eggs, larvae, protonymphs, deutonymphs, adult females and adult males, indicating a role throughout the life-cycle. This result was consistent with the transcript patterns of CHS1 in other insects and mites, such as *Sogatella furcifera* [19], *Diaphorina citri* [27], *Sitobion avanae* [18], and *Panonychus citri* [13]. Moreover, numerous previous studies in arthropods have indicated that mRNA expression of CHS1 is essential for the periodical molting process and CHS1 plays crucial roles in egg hatching, larval survival and adult fecundity [40]. Actually, *T. cinnabarinus* has a complex life history containing five distinct life stages. Like other arthropods, *T. cinnabarinus* develops and grows by ecdysis; this molting process involves the synthesis of a new exoskeleton by epidermal cells and partial degradation of the old exoskeleton. However, the peak expression level of CHS1 was different in various arthropods throughout their lifetime. A previous study by Zhang et al. indicated that the highest expression was observed in adults but the lowest in eggs of the oriental migratory locust (*Locusta migratoria manilensis*) [9]. In grain aphid *Sitobion avanae*, mRNA expression level was the highest during the first-instar stage and was more than twice that what was obtained during the second-, third- or fourth-instar and adult stages [18]. The recent study performed by Ullah et al. revealed that mRNA transcription of CHS1 of *Aphid gossypii* was highest in the first-instar nymph [38]. In the present study, the peak expression level of *TcCHS1* was detected in the eggs. Therefore, we speculated that *TcCHS1* may play an essential role in chitin biosynthesis and participate in the growth and development of spider mites. 

Current pests control primarily applies various chemical pesticides. BPUs are known to be effective against insect and tetranychid mite pests [28]. Mahdy et al. found that the percentages of fifth nymphal instars of the desert locust *Schistocerca gregaria* treated with different concentrations of lufenuron failed to ecdysis to adults significantly increased compared with the control [21]. After *D. citri* nymphs were exposed to diflubenzuron for 48 h, the cumulative mortality sharply increased [27]. In diflubenzuron bioassay, our results revealed that it had higher toxicity against eggs than larvae. Additionally, diflubenzuron exposure exhibited a negative effect on egg and larval survival of *T. cinnabarinus*. Admittedly, the egg is the beginning of all developmental stages in spider mites and the successful hatching of eggs could directly affect the age distribution in spider mite populations. Moreover, the proportion of eggs was higher than that of the other stages of tetranychid mite species [41]. A previous study concerning stable stage distributions in spider mite populations by 25 separate life tables of *Tetranychus urticae* revealed that all of the stage distributions were quite similar, averaging roughly 66% eggs, 26% immatures (larvae and nymphs) and 8% adults [42]. Dover et al. reported that the stage distribution model of *Panonychus ulmi* populations in an apple orchard was 60% egg, 30% immatures (14% larva, 8% protonymph and 8% deutonymph) and 10% adults (2% pre-ovipositing adult female and 8% adult) [41]. Therefore, we believe that diflubenzuron may be used to effectively control the *T. cinnabarinus* population. 

At the molecular level, several studies were focused on the mode of action about diflubenzuron, and the majority of the results demonstrated that diflubenzuron targeted the chitin synthase, which affects chitin biogenesis of the cuticle metabolism in arthropods [13]. However, the specific molecular mechanisms are still unclear. In the present study, different sublethal concentrations of diflubenzuron enhanced the mRNA expression levels of chitin synthase 1 in *T. cinnabarinus*. It is similar to the results of previous studies [39]. In third-instar larvae of *Anopheles quadrimaculatus*, the transcriptional levels of CHS were significantly increased at 100 and 500 ug/L diflubenzuron concentrations [7]. When the fourth-instar nymphs of brown citrus aphid *Toxoptera citricida* were exposed to 500 mg/L diflubenzuron, the mRNA expression level of CHS was 1.49-fold higher than the control [39]. Similarly, Lu et al. revealed the mRNA expression level of the CHS of fifth-instar nymphs of *Diaphorina citri* was significantly increased after diflubenzuron exposure [27]. These increasing CHS mRNA expression levels may result from the existence of a feedback regulatory mechanism to compensate for the enzyme inhibited by diflubenzuron [39]. However, some studies also indicated no effect of BPUs on CHS expression, such as in *Plutella xylostella* [29], *Drosophila melanogaster* [23] and *Tribolium Castaneum* [28]. Therefore, further studies of the proteomic and genomic levels are necessary to elucidate the effects of BPUs on the molecular regulatory mechanism of chitin biosynthesis in arthropods. 

## 5. Conclusions

The full-length cDNA of chitin synthase 1 (*TcCHS1*) from *T. cinnabarinus* was cloned and characterized in this study, and it belonged to the *CHS1* gene family by phylogenetic analysis. Additionally, *TcCHS1* was expressed at all different developmental stages. In addition, sublethal concentrations of diflubenzuron-treated *T. cinnabarinus* eggs and larvae resulted in increased mortality rates and elevated mRNA expression levels. Our results demonstrated that *TcCHS1* was essential for growth and development, and diflubenzuron exposure affected chitin metabolism. Further research is necessary to explore the biological function of *TcCHS1* based on the RNAi method. 

## Figures and Tables

**Table 1:** Primers and their sequences used in the present study.

Primer Name	Primer Sequence (5′-3′)	Purpose of Experiment	Amplicon Lengths
TC-F1	AYTGYTTYTGYTTYATHCCKGG	Short fragment	500 bp
TC-R2	TASAKACCCADATYATKCCRTG		
TC-F2	TYYTYATHTCANTHGGKTGGTG		
TC-R1	CKYTGRATRCARATYTTRCAAGC		
TC-F3	CYATAYGTTCARTAYGAYCARG	Short fragment	500 bp
TC-R4	ARRCARTGRAAYTCTTGYGGAT		
TC-F4	CYTAYACWCAYTGYCCWGARGG		
TC-R3	AYTCTTGYGGRTGMARGARTGC		
TC-CHS-F1	TGGGTGGTGGGAAAACTACATTG	Long fragment	1800 bp
TC-CHS-R2	GCTATGAAAAATACGGCAGACGG		
TC-CHS-F2	AATCTGCCTTCACTTCTCACA		
TC-CHS-R1	AAGAAAAATGGTTCCTGGTCC		
TC-5CHS-R1	GAGTATTTTGTGAGAAGTGAAGGCAG	5′RACE	750 bp
TC-5CHS-R2	TCAATGTAGTTTTCCCACCACC		
TC-3CHS-F1	CAAATGGCTGAAGATGGTGTTAC	3′RACE	1800 bp
TC-3CHS-F2	ATCTGGGTCTTTTGTAGTGGCGG		
UPM long	CTAATACGACTCACTATAGGGCAA AGCAGTGGTATCAACGCAGAGT	Universal primer	
UPM short	CTAATACGACTCACTATAGGGC		
NUP	AAGCAGTGGTATCAACGCAGAGT		
TC-CHS-F	CCAGTTGGTAGCGGTCTCA	RT-qPCR analysis	200 bp
TC-CHS-R	GCCTCATCGGATCTTGTCGT		
β-actin-F	CAGCCATGTATGTTGCCATC	Reference gene	200 bp
β-actin-R	AAATCACGACCAGCCAAATC		

F: forward primer; R: reverse primer.

**Table 2:** Toxicological parameters of diflubenzuron against eggs and larvae from *T. cinnabarinus*.

Stages	LC-P Equation	LC_50_ Median Lethal Concentration (mg/L)	95% Confidence Interval	Correlation Coefficient R	X^2^
Larvae	y = −1.582 + 1.303x	16.373	12.457–20.397	0.909	7.951
Eggs	y = −1.516 + 1.264x	15.825	11.895–19.855	0.926	6.242

**Table 3:** The corresponding sublethal concentrations of diflubenzuron against eggs and larvae of *T. cinnabarinus*.

Concentration (mg/L)	Eggs	Larvae
LC10	1.534 (0.662–2.671)	1.700 (0.768–2.887)
LC20	3.417 (1.815–5.226)	3.700 (2.034–5.553)
LC30	6.089 (3.734–8.534)	6.481 (4.078–8.955)
LC40	9.976 (6.853–13.095)	10.464 (7.322–13.593)
LC50	15.825 (11.895–19.855)	16.373 (12.457–20.397)

**Table 4:** Percent survival of eggs and larval survival rate of *T. cinnabarinus* eggs treated with sublethal concentrations of diflubenzuron.

Treatments	Number of Eggs	Survival Rate of Eggs (%)
LC50	148	48.16c
LC40	136	52.31c
LC30	132	72.13b
LC20	141	77.65ab
LC10	126	89.37a
CK	122	94.66a

**Notes**: CK served as the control group. The data are expressed as means ± SE of three replicates and subjected to one-way ANOVA followed by Duncan’s multiple range test. Different letters above each bar indicate a statistically significant difference at *p* < 0.05; same lowercase letters indicate non-significant changes.

## Data Availability

Data sharing not applicable.
